# Can Selenium and Molybdenum Restrain Cadmium Toxicity to Pollen Grains in *Brassica napus*?

**DOI:** 10.3390/ijms19082163

**Published:** 2018-07-24

**Authors:** Marwa A. Ismael, Ali Mohamed Elyamine, Yuan Yuan Zhao, Mohamed G. Moussa, Muhammad Shoaib Rana, Javaria Afzal, Muhammad Imran, Xiao Hu Zhao, Cheng Xiao Hu

**Affiliations:** 1 Key Laboratory of Arable Land Conservation (Middle and Lower Reaches of Yangtze River), Ministry of Agriculture, Huazhong Agricultural University, Wuhan 430070, China; maf02@fayoum.edu.eg (M.A.I.); elyoh@hotmail.fr (A.M.E.); yuanyuanzhao666@gmail.com (Y.Y.Z.); MohamedGomaa_Ali@agr.asu.edu.eg (M.G.M.); muhammadshoaib@webmail.hzau.edu.cn (M.S.R.); juvaria_afzal@outlook.com (J.A.); imrangorayauaf@yahoo.com (M.I.); xhzhaowhu@163.com (X.H.Z.); 2 Hubei Provincial Engineering Laboratory for New-Type Fertilizers, Huazhong Agricultural University, Wuhan 430070, China; 3 Botany Department, Faculty of Science, Fayoum University, Fayoum 63514, Egypt; 4 Soil and Water Research Department, Nuclear Research Center, Egyptian Atomic Energy Authority, Abou Zaabl 13759, Egypt

**Keywords:** oilseed rape (*Brassica napus*), foliar fertilizers, heavy metal stress, molybdenum, selenium, pollen grains, metal transporters

## Abstract

Cadmium (Cd) is highly toxic, even at very low concentrations, to both animals and plants. Pollen is extremely sensitive to heavy metal pollutants; however, less attention has been paid to the protection of this vital part under heavy metal stress. A pot experiment was designed to investigate the effect of foliar application of Se (1 mg/L) and Mo (0.3 mg/L) either alone or in combination on their absorption, translocation, and their impact on Cd uptake and its further distribution in *Brassica napus*, as well as the impact of these fertilizers on the pollen grains morphology, viability, and germination rate in *B. napus* under Cd stress. Foliar application of either Se or Mo could counteract Cd toxicity and increase the plant biomass, while combined application of Se and Mo solutions on *B. napus* has no significant promotional effect on plant root and stem, but reduces the seeds’ weight by 10–11%. Se and Mo have decreased the accumulated Cd in seeds by 6.8% and 9.7%, respectively. Microscopic studies, SEM, and pollen viability tests demonstrated that pollen grains could be negatively affected by Cd, thus disturbing the plant fertility. Se and Mo foliar application could reduce the toxic symptoms in pollen grains when the one or the other was sprayed alone on plants. In an in vitro pollen germination test, 500 μM Cd stress could strongly inhibit the pollen germination rate to less than 2.5%, however, when Se (10 μM) or Mo (1.0 μM) was added to the germination medium, the rate increased, reaching 66.2% and 39.4%, respectively. At the molecular level, Se and Mo could greatly affect the expression levels of some genes related to Cd uptake by roots (*IRT1*), Cd transport (*HMA2* and *HMA4*), Cd sequestration in plant vacuoles (*HMA3*), and the final Cd distribution in plant tissue at the physiological level (*PCS1*).

## 1. Introduction

Cadmium (Cd) as one of the most toxic metals to both vertebrates and vascular plants is widespread in soil, water, and the atmosphere due to industrial emissions, sewage sludge, as well as phosphate fertilizers applications, and public waste disposal containing Cd [1,2]. Its presence in soil, water, and even the atmosphere can cause serious problems to all organisms, and its bioaccumulation in the food chain can be highly dangerous [3]. Although Cd is not essential for plant growth, it is readily absorbed by the plants and translocated to the edible parts where it is accumulated at high levels. Cd interferes with many cellular functions leading to retardation of plant growth, leaf chlorosis and a decrease in photosynthesis rate, diminishing water and nutrient uptake, which might ultimately result in the plant death [4]. In addition, it can cause oxidative stress and alter the functionality of membranes and the nitrogen metabolism [5].

*Brassica napus* is widely planted as a Cd-hyperaccumulator [6]. In *B. napus*, Cd is reported to cause suppressed growth, decrease in the photosynthetic pigments content, as well as the activities of antioxidant enzymes, and increases in malondialdehyde and reactive oxygen species (ROS). In addition, it severely damages the leaf and root tip cell [7]. Due to its high mobility, Cd can be transferred to the reproductive tissues of the plant, such as flowers and pollens to affect their development and impair seed formation. Based on literature survey, Cd^2+^, Cu^2+^, and Hg^2+^ showed the highest toxicity towards the in vitro pollen germination and pollen tube growth in *Lilium longiflorum* [8]. In addition, Cd^2+^ was the only one out of the screened metals which was found to cause intracellular interactions and affect the organelles distribution within the pollen tip regions [9,10]. Several studies indicated that cadmium strongly inhibits in vitro pollen germination and pollen tube growth [11,12,13]. Cd also negatively affects plant reproduction by inhibiting pollen germination and ovule growth and, therefore, induces invalid flowers and shriveled grains [14].

Selenium (Se) is considered an essential nutrient for humans and animals [15,16], however, not for some other organisms, including plants. At low dosages, Se exerts diverse beneficial effects on different plant physiological processes leading to enhanced plant growth and high yields [17]. The use of selenium in the form of foliar spray or base fertilizer is well documented to increase its content in the edible parts of the plants. It can also mitigate the negative effects caused by variety of environmental stresses [18]. Se has been used to protect plants subjected to Cd [19], though the mechanism of its action is not completely clear, however, the regulation of ROS, antioxidants, Cd-uptake, and its further translocation, Cd-storage in non/less-toxic forms, as well as the recovery of the photosynthetic system and reconstruction of cell membranes and chloroplasts, has been proved to be involved in the protection process [18].

Molybdenum (Mo) is one of the micronutrients required by plants as well as animals in very small amounts for normal growth. It is principally used in the production of “molybdo-enzymes” that control various plant functions. In plants, Mo deficiency negatively affects some plant activities such as photosynthesis, root growth, and some nitro-enzymes [20,21]. In addition, it affects flowering and pollen production capacity, the grain size and pollen germination, thus, the quality and production of crops are negatively affected [22]. Foliar applications of Mo are proved to be a very beneficial source of Mo, even more effective than soil applications, particularly for acidic soils [23], or under dry conditions [24]. Application of Mo could increase growth, photosynthesis, chlorophyll, and the antioxidative capacity of plants [25]. Molybdenum application enhances the salinity stress tolerance in *Brassica campestris* by increasing its capacity to eliminate active oxygen [26]. The potential of Mo to alleviate the acute toxicity of Cd in animals has been recognized a long time ago [27]. However, just recently, molybdenum has been reported to increase the phytoremediation ability of *Ricinus communis* L. for Cd removal and to alleviate Cd-toxic symptoms under Cd stress [28].

Heavy metals uptake and transport in plants are controlled by several types of metal transporters [29]. Cd uptake by plant root cells can happen via both passive and active transport. The active uptake of Cd by the plant roots is mediated by *ZRT* (zinc-regulated transporter) and *IRT* (iron-regulated transporter) [30,31]. In *Arabidopsis thaliana*, *AtIRT1* is a primary transporter for iron uptake and it also mediates Cd uptake by the plant roots [30]. Cd loads from symplasm into the xylem by heavy metal P_1B_-ATPases, such as *HMA2* and *HMA4* [32]. In addition, *HMA3* participate in heavy metal ion transport and detoxification and it is mainly involved in the vacuolar storage of Cd [33]. It also plays a role in Cd sequestration into root or leaves vacuoles, thus controlling Cd reaching plant seeds and enhancing the plant tolerance to Cd [34]. Phytochelatins (PCs) are small cysteine-rich peptides, that are synthesized in plants using glutathione as a substrate in a catalyzed process mediated by phytochelatin synthase *PCS*. Cd exposure also induces the expression of phytochelatin synthase and, thus, increases the level of phytochelatins which can make a complex with Cd (PCs–Cd complex) then it is sequestered into vacuoles for Cd detoxification [35]. Thus, overexpression of *PCS*, especially *PCS1* was shown to confer Cd tolerance and control Cd accumulation in seeds.

In most plants, fruit formation and seed-set necessitate effective pollination, and the understanding of pollen biology, including pollen viability and pollen tube growth is required to raise the plant productivity. In many plants, the reproductive tissues are more sensitive to Mo and Se deficiency than vegetative tissues, and such deficiency may affect pollen fertility. In spite of the well documented protective role of Se and Mo, there is no available data about the possible extension of this role to the reproductive organs, either in vivo or in vitro. In addition, Mo and Se deficiency are very popular in arable land of South China, particularly in the area where *B. napus* grows. Thus the objectives of this present study are to (i) evaluate the effect of foliar application of Mo and Se, either alone or in combination, on Cd uptake and translocation in *B. napus* which is the third-largest source of vegetable oil in the world, and it is also used as a vegetable [36], (ii) investigate whether Mo and Se can restrain transferring of Cd to reproductive parts such as pollens and seeds, (iii) explore the effect of applying Se and Mo on pollen morphology and viability in Cd-stressed *B. napus*, and (iv) finally to study the protective role of Se and Mo at the molecular level via studying some metal transporters that might play a role in Cd-uptake and translocation.

## 2. Results

### 2.1. Plant Biomass

**Figure 1:**
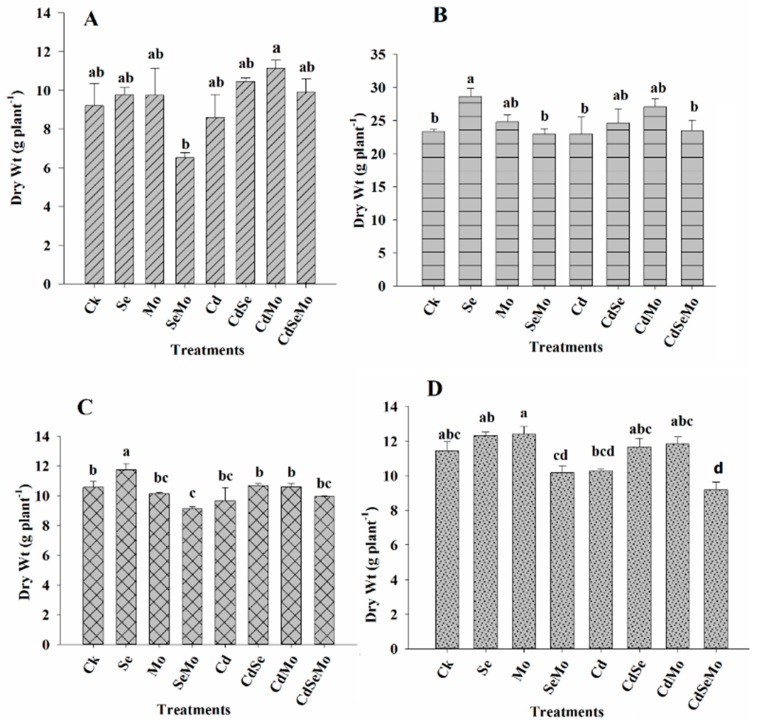
Dry biomasses of *B. napus* grown under different experimental conditions. Dry weight of root (**A**), stem (**B**), glume (**C**), and seed (**D**) of oilseed rape treated with foliar spray of (1 mg/L) Se and/or (0.3 mg/L) Mo in absence or presence of Cd stress (5 mg/kg). All data show the means ± SD of four replicates. Different letters within a column indicate significant (*p* < 0.05) differences between treatments.

The variation in dry mass of root, stem, glume, and seed of *Brassica napus* under Cd stress is presented in Figure 1. Under normal conditions, without Cd stress, foliar spray of Se and Mo as fertilizers improved root, stem, glume, and seed total biomasses compared to the control. However combined Se and Mo significantly reduced the overall plant biomass. Cd application has negatively affected plant growth and reduced root, stem, glume, and seed dry weights by 6.52%, 1.65%, 8.45%, and 10.23% respectively, compared to the control. Under Cd stress, foliar application of Mo alleviated the Cd toxicity and considerably increased the plant biomass by 29.56%, 17.79%, 9.41%, and 15.15% for root, stem, glume, and seeds, respectively, compared to Cd application alone. Although Se foliar application enhanced plant growth and increased plant dry weight, compared to Mo application, Se effect was lower. Combined application of Se and Mo under Cd stress has no significant promotional effect on plant root and stem, however, significantly decreased glume and seed dry mass. These results suggest that Se and Mo separately enhance plant growth and could alleviate the toxic effect of Cd.

### 2.2. Cd Concentration and Accumulation

**Figure 2:**
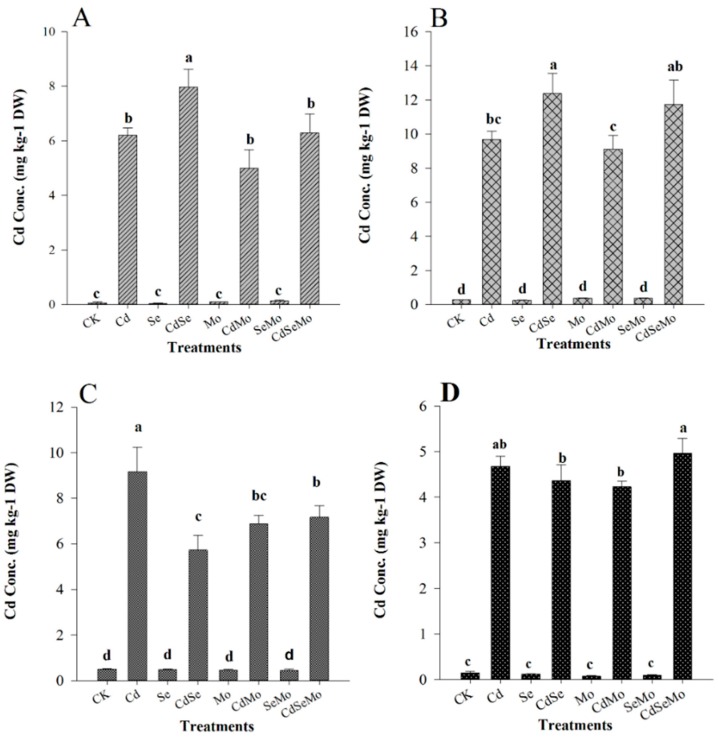
Concentrations of Cd in *B. napus* tissues. Cd concentration in root (**A**), stem (**B**), glume (**C**), and seed (**D**) of oilseed rape treated with foliar spray of (1 mg/L) Se and/or (0.3 mg/L) Mo in absence or presence of Cd stress (5 mg/kg). All data show the means ± SD of four replicates. Different letters within a column indicate significant (*p* < 0.05) differences between treatments.

The impact of selenium and molybdenum on cadmium uptake and its distribution within oilseed rape plant was investigated. The total concentrations of cadmium, selenium, and molybdenum were determined in roots, stems, and fruits of oilseed rape and are presented in Figure 2. In one hand, Cd concentrations in roots decreased with Mo treatment by 19.35% (Figure 2A), by the same way that of stem was reduced by 5.71% (Figure 2B), suggesting that Mo inhibited Cd accumulation in roots and consequently reduced heavy metal toxic effects in stems. However, when Cd was supplied simultaneously with Se, its accumulation in roots was even more pronounced than when supplied separately and consequently increase heavy metals accumulation in roots and stems by 28.43% and 27.92%, compared with the control (Figure 2B). These results suggest that Se enhance the plant’s tolerance against Cd and leads to a considerable increase of Cd on the edible part of the plants. Surprisingly, applying both Se and Mo together has no significant effect in Cd root concentration, however, in stems it is slightly increased compared to control.

In the other hand, the trend of Cd accumulation in glume and seeds was slightly different from that of root and stem. In glume, separate Mo and Se foliar fertilization has significantly decreased Cd concentration by 24.9% and 43.5%, respectively, compared to that of Cd treatment alone (Figure 2C). In seeds, the differences of Cd concentrations between all the treatments are not as high as in glume, however, Cd concentration decreased with spraying Mo or Se separately and increased when Mo and Se were used together (Figure 2D). Considering the total Cd accumulated in the fruits (glume and seeds together), the sequence of Cd concentration follows the trend; Cd > Cd + Mo + Se > Cd + Mo > Cd + Se with 13.83, 12.13, 11.09, 10.07 mg Cd/kg DW, respectively. This result highlights that Se or Mo spray significantly decreases the total Cd reaching the plant fruit, though the amount of Cd accumulated in seeds is higher in the case of Se as compared to Mo.

### 2.3. Se and Mo Contents in Oilseed Rape

**Figure 3:**
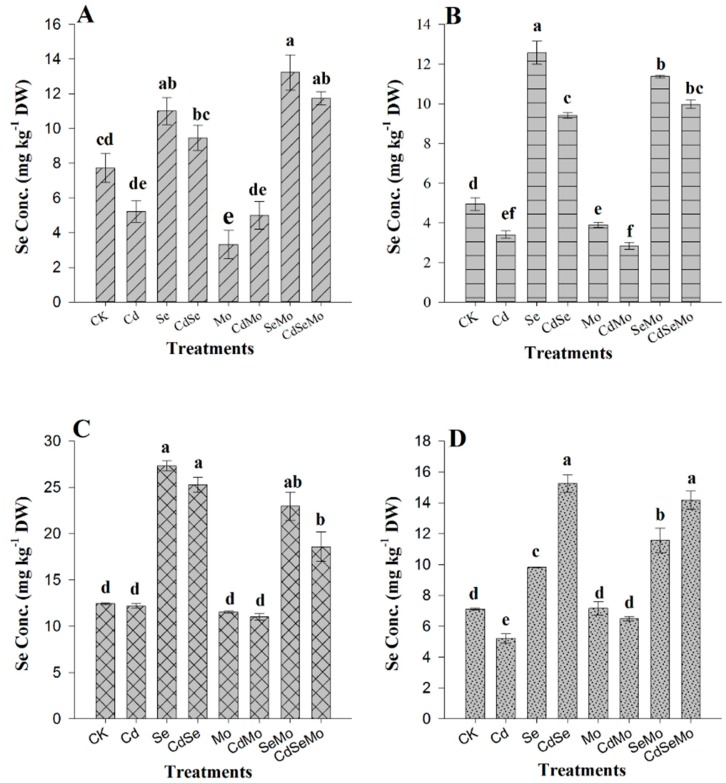
Concentrations of Se in *B. napus* tissues under different treatment conditions. Se content in root (**A**), stem (**B**), glume (**C**), and seed (**D**) of oilseed rape treated with foliar spray of (1 mg/L) Se and/or (0.3 mg/L) Mo in absence or presence of Cd stress (5 mg/kg). All data show the means ± SD of four replicates. Different letters within a column indicate significant (*p* < 0.05) differences between treatments.

**Figure 4:**
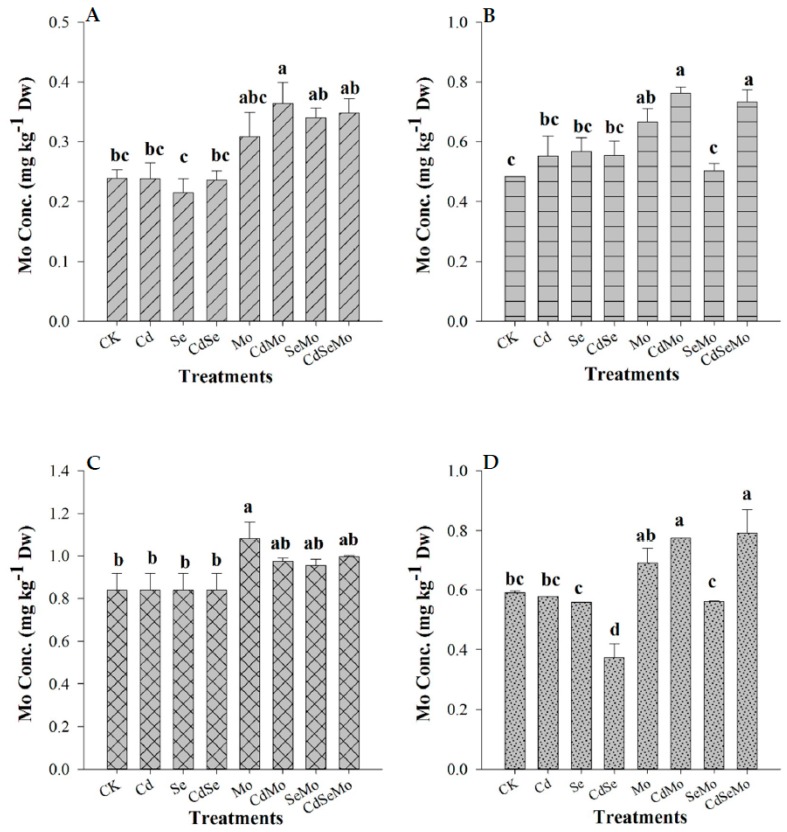
Concentrations of Mo in *B. napus* tissues under different treatment conditions. Mo content in root (**A**), stem (**B**), glume (**C**), and seed (**D**) of oilseed rape treated with foliar spray of (1 mg/L) Se and/or (0.3 mg/L) Mo in absence or presence of Cd stress (5 mg/kg). Bars indicate standard error (*n* = 4). Different letters indicate significant differences at (*p* < 0.05).

Se and Mo concentrations in overall plant tissues are presented in Figure 3 and Figure 4. Se, in all plant parts was significantly increased when it was applied as foliar spray following the order: Stem > glume > root > seed with 154.49%, 119.52%, 42.42%, and 37.92%, respectively, as compared to control. However, Se was not effectively translocated from shoots to roots, since a relatively lower increase of Se concentration (42.42%) was observed in roots as compared to stems (154.49%). Nevertheless, co-application of Mo (i.e., in Mo + Se treatments) enhances Se transport from shoot to root which was increased by 20.24% in root and decreased by 9.17% in shoot. In addition, foliar spray of Mo significantly increases Se content in seeds. In those treatments where Mo was applied as a foliar spray, its content significantly increased in the overall plant. However, in other treatments (CK, Cd, Se, Cd + Se), no difference in Mo concentration in all plant parts was denoted, except in Cd + Se where Mo content was significantly decreased in seeds.

Soil Cd has an impact on Se accumulation since it decreases Se concentration in stems, roots, and glumes by 25.21%, 13.98%, and 7.4%, respectively, compared to Se alone. In addition, it resulted in a substantial increase in Se content of seed by 55.29% as compared to Se only. When Mo was sprayed with Se, under cadmium stress, Se concentration in both root and seed was not affected compared with the plants supplemented with Se alone, however, it significantly decreased in stems, and glumes. Interestingly, Cd stress considerably increased the plant Mo uptake on foliar spraying, thus increasing Mo content in roots, stems, and seeds compared with those treatments supplemented with Mo alone (Figure 4). Se also affected the plant Mo uptake, since in the Se + Mo treatment, the accumulation of Mo in stems, glumes, and seeds was significantly decreased, but the root Mo content slightly increased. These results suggest that Se decreases Mo translocation rate from shoots to roots, however, in Cd-polluted soil, Mo concentration increased in roots whether Se was applied or not.

### 2.4. Anther and Pollen Grains Morphology

#### 2.4.1. Microscopic Studies

**Figure 5:**
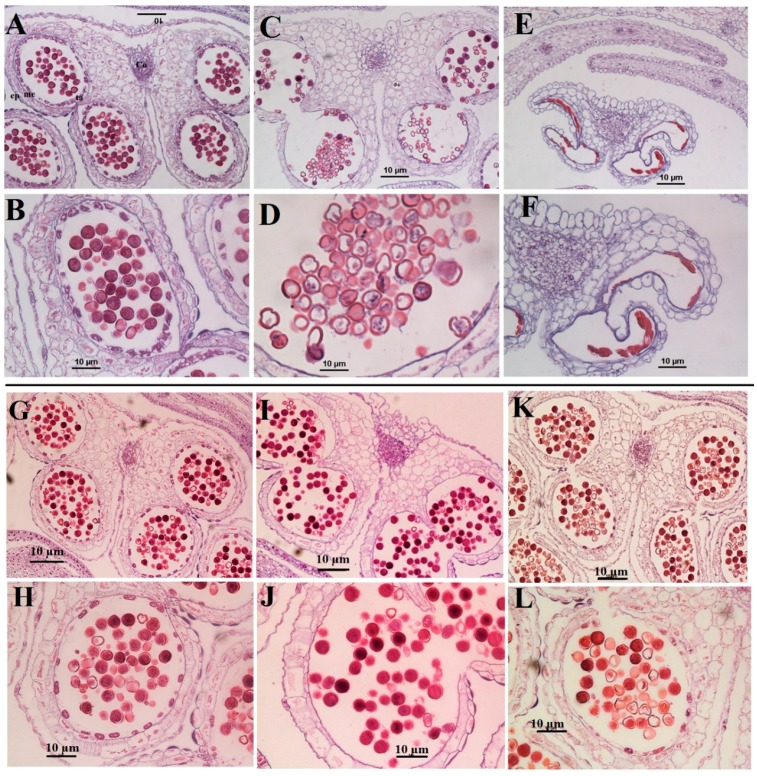
Transverse sections through distinct anther of *B. napus*. Collected from; CK (**A**,**B**), Cd-treated plants (**C**–**F**), Cd + Se (**G**,**H**), Cd + Mo (**I**,**J**), Cd + Se + Mo (**K**,**L**). Plants were treated with foliar spray of Se (1 mg/L) and/or Mo (0.3 mg/L) in absence or presence of Cd stress (5 mg/kg). Pollen grains are spherical in both polar and equatorial view, with dense cytoplasm and a prominent centrally located nucleus. **co**: connective, **ep**: epidermis, **me**: middle layer, and **ta**: tapetum. Bar is 10 μm.

Pollen structure was determined for samples grown in polluted soil under all the experimental conditions in our study and compared to the control, as presented in Figure 5. In normal plants, collected from control (CK), the anther basically consisted of four pollen sacs which occupied the bulk of each anther. Under Cd stress, plants displayed clear morphological anomalies during this stage. Pollen sacs suffered the formation of irregularly shaped microsporocytes (Figure 5C,D) and the number of pollen grains per pollen sac were significantly decreased compared to the control. In some cases, anther growth was even incomplete and became smaller than the control probably due to high stability of the anther wall as a defense mechanism to minimize Cd reaching the pollen grains. In other cases, Cd rendered mature anthers sterile; their exines became completely crushed and they are symbolized by a dense mass in each locule of the anther at flower anthesis (Figure 5E,F). Based on these results, it can be concluded that the anther development in *Brassica napus* was seriously affected by Cd. Yet, when Se or Mo was applied individually in Cd-polluted plants the pollen grains and anther seem to recover the abnormalities caused by Cd following the decrease in Cd concentration observed in seeds (Figure 5G–J). However, foliar application of both Se and Mo together on leaves resulted in more abnormalities in anther and pollen grains (Figure 5K,L).

#### 2.4.2. Scanning Electron Microscopy

**Figure 6:**
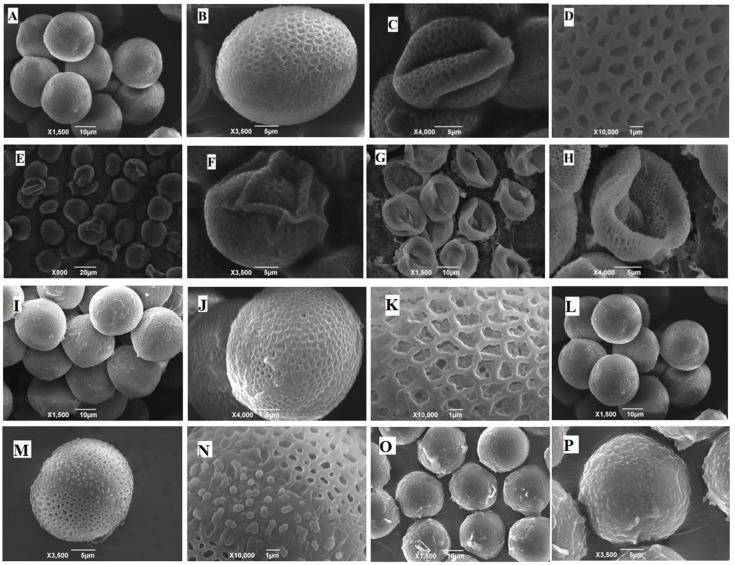
Scanning electron micrographs of *B. napus*’ pollen grains. Control (**A**–**D**), plants treated with Cd (**E**–**H**), Cd + Se (**I**–**K**), Cd +Mo (**L**–**N**), and Cd + Se + Mo (**O**,**P**). Plants were treated with foliar spray of Se (1 mg/L) and/or Mo (0.3 mg/L) in absence or presence of Cd stress (5 mg/kg).

The pollen grains were further observed by scanning electron microscopy (SEM) to investigate the effects of heavy metals on their morphology. Results from this observation are shown in Figure 6. The morphology of pollen grains was found presenting colpate, prolate, and reticulate sculpture. Pollen grains from Cd-stressed samples displayed thicker exine and raised muri than pollen grains of control plants. In addition, some pollen became sterile devoid of tryphine in the tectal cavities. Finally, an unusual adhesion of pollens collected from Cd-stressed samples was also detected. Similar to microscopic studies, SEM confirmed that in Cd contaminated soil, Se and Mo foliar application could release the toxic symptoms in pollen grains when they are being sprayed alone. However combined usage of them presents negative effect on pollen grains morphology.

### 2.5. Pollen Grains Fertility by I_2_/KI (Iodine) Staining

**Figure 7:**
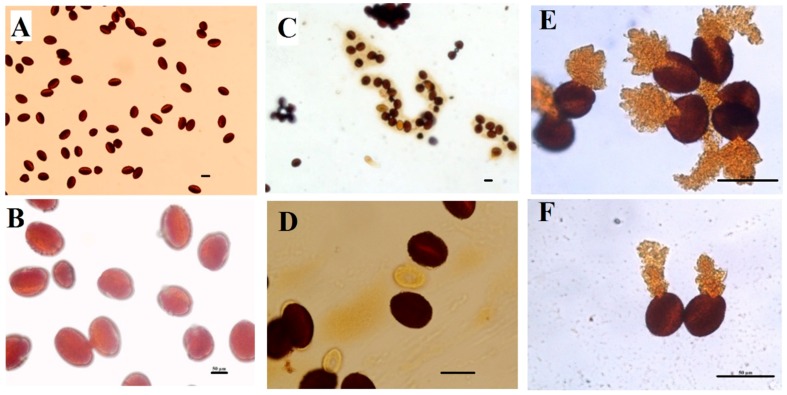
Pollen fertility of *B. napus*’ pollen grains. I_2_/KI staining for starch, normal mature pollen grains containing starch granules stain black or dark brown; however, the immature pollen grains appear orange to red; CK (**A**,**B**) and Cd-treated plants (**C**–**F**). Pollen from control have uniform shape, but Cd treated plants sometimes have burst pollen. Pollens were collected from plants treated with foliar spray of Se (1 mg/L) and/or Mo (0.3 mg/L) in the absence or presence of Cd stress (5 mg/kg). Scale bar: 50 μm.

**Figure 8:**
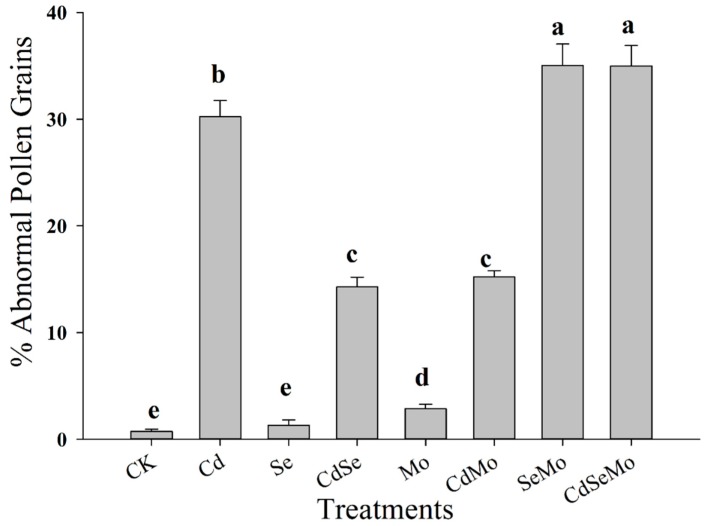
Effect of cadmium, selenium, and molybdneum on % of abnormal pollen grains in *B. napus*. Pollens were collected from plants treated with foliar spray of Se (1 mg/L) and/or Mo (0.3 mg/L) in the absence or presence of Cd stress (5 mg/kg). Different letters indicate significant differences at (*p* < 0.05).

Pollen viability refers to the ability of pollen grains to complete post-pollination process and to achieve fertilization. The viability of pollen grains collected from all the treatments was checked and the results are shown in Figure 7. In this test, mature pollens are easily distinguished from the immature ones by color, while normal pollens (mature) stain black to dark brown; abnormal ones (immature) stain orange to red. Pollens from control plants have uniform shape, however, burst pollens are sometimes observed in those samples collected from plants exposed to Cd stress (Figure 7E,F). In addition, viability percentages of pollen grains were reduced proportionally under Cd stress. However, individual application of Se and Mo reduces the number of abnormal pollen grains, while combined application of both in the presence or absence of Cd increases the percentage of the abnormal pollen (Figure 8). This result shows that foliar application of Se and Mo together at these concentrations has a toxic effect on pollen grains similar to that of Cd.

### 2.6. Pollen Germination and Pollen Tube Growth In Vitro

**Figure 9:**
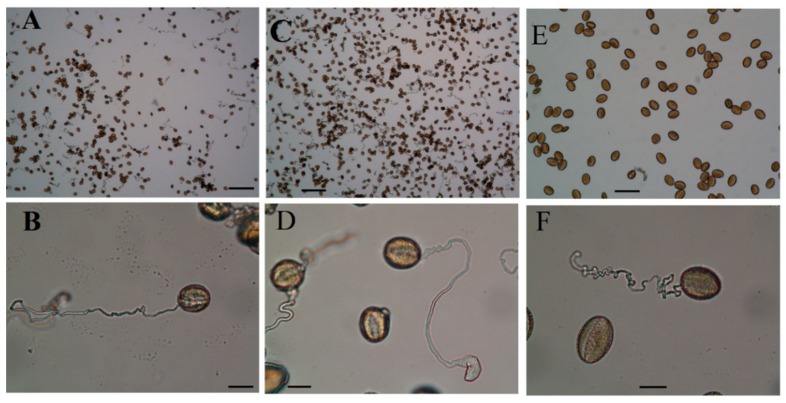
Effect of Cd on in vitro germination of pollen grains in *B. napus*. Control (**A**,**B**), 100 μM cadmium-treated sample (**C**,**D**), 500 μM cadmium-treated sample (**E**,**F**), pollens are germinated in a culture medium contains polyethylene glycol 4000 (20%) and sucrose (5%). Scale bar: 20 μm.

**Figure 10:**
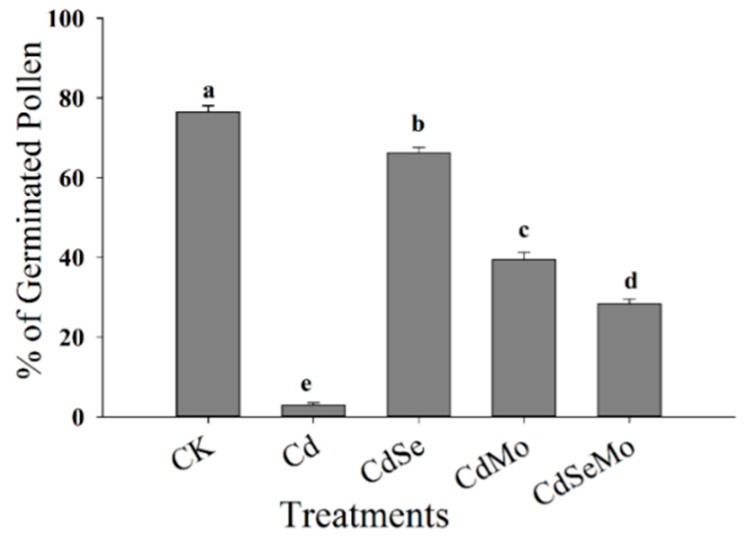
In vitro germination (%) of pollen grains under Cd stress. Pollens are germinated in a culture medium contains polyethylene glycol 4000 (20%) and sucrose (5%) under 500 μM Cd in the presence of Se (10 μM) and/or (1 μM) Mo. Different letters indicate significant differences at (*p* < 0.05).

The effect of cadmium ions on the in vitro pollen germination rate of *B. napus* was investigated by light microscopy. In vitro pollen germination of *B. napus* under control conditions is characterized by around 75% of all cultured pollen grains that had germinated, indicating a high potency of the pollen grains used in the present study and the suitability of the used germination medium. To determine the toxic levels of Cd on pollen grains, a series of experiments were conducted where pollen grains were allowed to germinate in culture media containing cadmium at different concentrations, and the results showed that low Cd concentrations up to 100 μM enhance the pollen germination rate. However, the pollen tube was sometimes noticed to have a swollen tip (Figure 9C,D), which was absent in the control. Pollen tubes of control experiments tend to grow straight with only an occasional change in direction. In contrast, in germination medium containing Cd, even at the low concentrations which displayed an increase in pollen germination rate; the pollen tubes grow extremely contorted. At concentrations higher than 100 μM, cadmium treatment inhibited pollen germination and tube growth in a dose-dependent manner. In the second set of experiments, three concentrations of Se and Mo were independently checked to reach the best concentration that would positively affect the pollen germination rate and pollen tube growth. We observed that high concentrations of Se (20 μM) or Mo (10 μM) have significantly inhibited the pollen germination rate and affect the pollen tube growth. From this preliminary experiment, we have determined that, for *B. Napus*, 10 μM Se and 1 μM Mo are the optimum stimulatory concentration for in vitro pollen grain germination, while 500 μM of Cd is highly toxic and prevents the pollen germination. Thereafter, pollen was germinated under Cd stress of 500 μM in the presence of Se (10 μM) and/or Mo (1 μM). Germination rate decreased in Cd treatment to less than 2.5%, but it increased when Se or Mo was added to the germination medium to reach 66.2% and 39.4%, respectively (Figure 10). When pollens were germinated in a medium containing, both selenium and molybdenum, the improvement in the germination rate was much lower than in the case of either Se or Mo alone.

### 2.7. Relative Expression Analysis of Genes Related to Cd Uptake, Transport, and Detoxification

**Figure 11:**
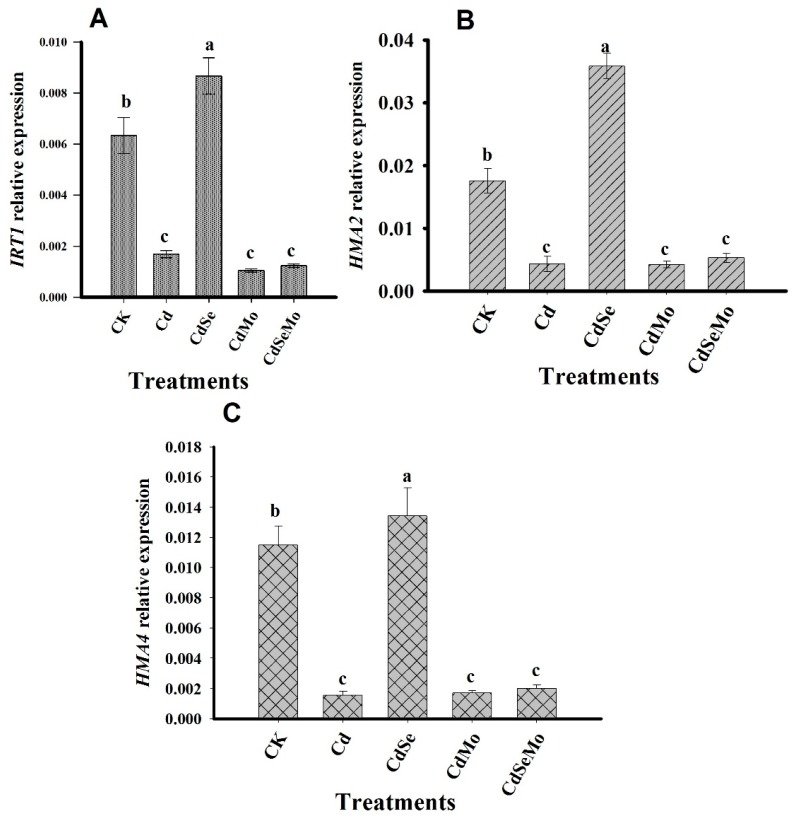
Expression profiles of *IRT1* (**A**), *HMA2* (**B**), and *HMA4* (**C**) in roots of *B. napus*. Vertical bars indicate standard error of each mean (*n* = 3). Different letters within a column indicate significant differences between treatments at (*p* < 0.05). The relative expression levels were calculated by comparing the cycle thresholds (CTs) of the target genes with that of the reference gene *BnActin* using the 2^−ΔΔ*C*t^ method.

To investigate how Se and Mo application could control Cd-uptake and its further transport at the genetic level, we have examined the relative expression of several genes that might be involved in Cd uptake or detoxification such as *IRTI*, *HMA2*, *HMA3*, and *HMA4* in those treatments where plants were exposed to cadmium stress. However, studying those genes controlling Cd uptake and translocation could not completely explain the protective role of Se on pollen grains and the low Cd in seed. Therefore, another gene i.e., *PCS1* was considered which is known to provide Cd tolerance and control Cd accumulation in seeds. The expression of the *IRT1* gene in roots significantly increased (*p* < 0.01) in the presence of Se, as compared to Cd only. However, no significant differences were observed in *IRT1* expressions between other treatments, i.e., Cd, Cd + Mo, and Cd + Se + Mo (Figure 11A). *HMA2* and *HMA4* are expressed in the plant roots 10 times higher than in leaves. In roots, both of them showed the same trend where the genes are overexpressed in Cd + Se treatment. Mo treatments did not result in a significant change in the two genes expressions when applied alone, and it even down-regulates *HMA2* and *HMA4* expressions when it was amended with Se (Figure 11B,C).

**Figure 12:**
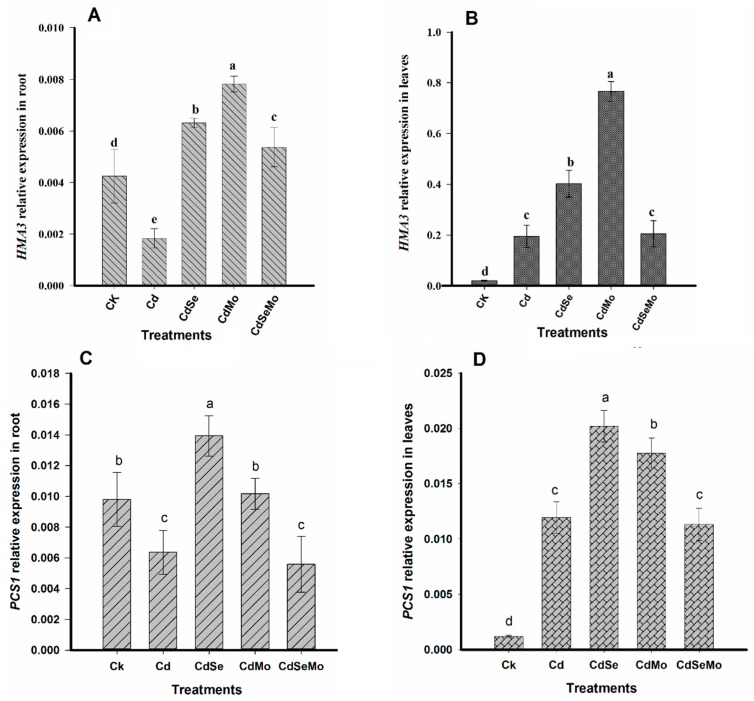
Expression level of *HMA3* in root (**A**) and leaves (**B**), and *PCS1* in root (**C**) and leaves (**D**) of *B. napus* vertical bars indicate standard error of each mean (*n* = 3). Different letters within a column indicate significant differences between treatments at (*p* < 0.05). The relative expression levels were calculated by comparing the cycle thresholds (CTs) of the target genes with that of the reference gene *BnActin* using the 2^−ΔΔ*C*t^ method.

Interestingly, *HMA3* was found to be strongly expressed in leaves hundred-fold higher than its expression in roots. In leaves, Mo application enhanced *HMA3* expression, while Se has no substantial effect on it (Figure 12A,B). These results interpret the high Cd content in the plant root and shoot in the presence of Se, which is due to the enhanced genes expressions responsible for Cd uptake, i.e., *IRT1* and Cd translocation from root to shoot, i.e., *HMA2* and *HMA4*. In case of Mo application, Cd uptake was reduced as observed by the low expression of *IRT1*. However, Cd translocation from root to shoot was not much affected, at least via *HMA2* and *HMA4* genes. Phytochelatins (PCs) are glutathione-derived peptides and its biosynthesis is considered one of the most important mechanisms that contribute to Cd accumulation and tolerance in plants. In our study, Cd exposure significantly stimulate the expression of *PCS1* in the plant leaves. Treating the plants with either Se or Mo enhanced the expression of *PCS1* over Cd-treatment alone, at a similar level. However, applying Se and Mo together did not enhance *PCS1* expression in Cd + Se + Mo treatment, when compared with Cd only (Figure 12C,D). This will lead to PC deficiency which in turn may cause Cd sensitivity, this could explain the negative effect of the combined application of Se and Mo when applied under Cd stress.

## 3. Discussion

It is well established that the production of plant biomass is an important indicator for evaluating plant tolerance to heavy metal stress, such as Cd [37]. In our study, Cd at 5 mg/kg soil reduced the growth of *B. napus* which consequently leads to the reduction of its tissues biomass. Plant growth inhibition by Cd may be due to the inhibition in cell division and its toxic effects on the function of some key enzymes involved in plant metabolism. Se or Mo foliar fertilization has improved the plant growth. Combined application of both Se and Mo solutions significantly reduced the overall plant biomass, regardless of Cd content (0 or 5 mg/kg). Several studies have demonstrated that Se can play diverse beneficial effects at low concentrations including growth-promoting activities of higher plants [38]. Molybdenum, as one of the micronutrients that plants need in very small amounts for normal growth, has also been proved to improve the growth parameters in different plants, such as *Helianthus annuus* L. [39,40], *Brassica napus* [41], and other plants. The stimulating effect of Mo on plant biomass might be due to its role as a cofactor for enzymes involved in nitrate metabolism (such as nitrate reductase and glutamine synthetase) and synthesis of amino acids and indole acetic acid [20]. Moreover, Mo application was found to enhance salinity stress tolerance in Chinese cabbage by increasing the photosynthesis rate and the ionic homeostasis adjustment [42]. In wheat, Mo supplementation increased the activities and transcripts of antioxidant enzymes, decreased H_2_O_2_ and MDA contents, and elevated NO production, implying that Mo-induced antioxidant defense may be related to NO signal [43]. Zhang et al., reported that when Se and Mo were used independently in the growth medium at 0.01 mg/L concentration, it slightly enhanced the growth of Chinese cabbage, however, at slightly higher Se concentration 0.1 mg/L, it significantly reduces the plant growth as indicated by more than 10% decrease in shoot DW, as compared to using Mo only [44]. The reduction in plant biomass in case of combined Se and Mo application can be due to more than one reason. Firstly, several reports have verified that both Mo and Se are transported through the plant via sulphate and phosphate transporters. Thus, these anions will compete with each other to interact with the transporters, which might affect the absorption of any of them, as well as sulfate transport [45,46]. Indeed, from our results we can observe that Mo concentration was significantly reduced in both stem and seed in Se + Mo treatment. This decrease in Mo concentration might affect many physiological processes, due to Mo incorporation into molybdopterin, an essential cofactor for enzymes involved in sulfite detoxification, purine catabolism, nitrate assimilation, and abscisic acid biosynthesis. In addition, Schiavon et al., reported that selenate and molybdate alter sulfate transport and assimilation, thus treating *Brassica juncea* with either Se or Mo led to a considerable decrease of sulphate uptake, this effect might be more pronounced in case of the combined application of both Se and Mo [47]. This was likely because selenate and molybdate competed with sulfate for access to the S metabolic pathway. In addition, selenium competition is known to cause a disruption of S metabolism and repressed synthesis of reduced S-containing compounds, such as Cys and GSH. Likewise, the mean shoot and root biomass of *Astragali* species was significantly reduced when treated with Se and Mo, regardless of whether the species are Se-hyperaccumulator or non-accumulator, with also reduced S assimilation in Se-non-accumulators [48].

Mo foliar fertilization decreased the plant Cd uptake as illustrated by its low concentration in root and stem. On the other hand, Cd content in both root and shoot was significantly increased with Se foliar application. Reports have shown that proper doses of Se can protect plants against the damage caused by heavy metals/metalloids and decrease its uptake and accumulation in the plant tissues, including Cd [49,50], Pb [51,52], Cr [53], Ni [54], Hg [55], Cu [56], As [57], and Sb [58]. The mechanisms of heavy metal detoxification by Se might be related to the inhibition of uptake and translocation of heavy metals from the roots to aboveground and/or the speciation transformation to nontoxic species. However, in some other cases, it was shown that Se can stimulate heavy metal accumulation such as Cd and Cu in wheat and pea [59], As in *Thunbergia alata* [60], Al in ryegrass [61], and Cd and Cu in the roots of *Sinapis alba* L. seedlings [62]. The increase of Cd accumulation in plant tissues, in our study, in case of Se foliar application is unlikely to be attributed to the improper dose of Se, since Se alone has a positive effect and enhance the plant growth and increase Se content in all plant tissues compared to control. It seems that heavy metal uptake and its translocation depend on several parameters such as plant species and cultivar, the used dose, application method (foliar spray or soil/solution additives), etc. In addition, the decrease of Cd reaching the plant fruit on applying Se, accompanied by the increase of Cd concentration in root and stem in the same treatment, suggest that the speciation conversion of Cd into nontoxic species and its sequestration into leaves vacuoles could be the reason of elevating Cd toxicity and enhancing the plant growth, which is confirmed from the study of metal transporters.

Foliar application of Se and Mo significantly increase their concentrations in all the plant parts, compared to controls, which means that *B. napus* can effectively take up Se and Mo through foliar application. Furthermore, when Mo is applied with Se, it enhances Se transport from shoot to root and increases Se concentration in root and reduce it in shoot. Zhang et al. [44] found an antagonistic relationship between Se and Mo uptake in Chinese cabbage when both were supplied in solution culture. Se concentrations in shoots and roots were significantly inhibited by application of Mo. In a recent study, application of Mo increased Se concentrations in pepper fruit, stem, leaf, and root in *Capsicum frutescens* L. [63], which is consistent with our observation. Mo uptake was found to increase under Cd stress, without a clear impact of Cd on the Mo translocation from shoot to root. Se application resulted in a decrease of Mo concentration in stem, glume, and seeds, but the root Mo content was increased. These results suggest that Se enhances Mo transfer from shoot to root which conforms to that found by others [45,46].

Microscopic studies revealed the appearance of several toxic symptoms on pollen grains and anthers collected from those plants grown merely under Cd stress which eventually affect *B. napus* fertility. Similar observations were reported in *Chenopodium botrys* L. grown in a mine area polluted with Fe, Mn, and Zn [64]. Lead was also reported to have toxic effects on the pollen grains in *Matricaria chamomilla* in an in vivo experiment; the toxic effects included decrease in the size and diameter of pollen sac wall, along with change of the spine shape [65]. On the other hand, some results showed that Cd exposure had no effect on in vivo pollen germination of *Pisum sativum* L. exposed to 7 mg/kg Cd in soil, however, the same study revealed that a Cd concentration at 10 μg/mL drastically inhibited the pollen germination rate in the in vitro experiment, reducing it to 11% compared to 90% in control [11]. Abnormalities development of pollen grains is not observed under heavy metal stress only, but other stress such as salinity cause the destruction of the anther wall and both the degeneration and production of abnormal pollen grains [66]. Results from SEM has also shown that pollen grain of *Brassica napus* has similar morphology to that of *Arabidopsis* [67]. Previous studies have shown that Se treatment could raise respiratory activity in leaves and flowers in *Brassica napus*, which may have contributed in turn to increase the seed production by 43% [11]. Several studies have shown a strong correlation between mitochondrial function and pollen viability [68]. Thus, it is expected that increased mitochondrial activity in the leaves and flowers would be reflected in pollen viability in the Se-treated plants. Additionally, Se was found to strongly counteract the ROS accumulation in olive pollen grains under draught stress and consequently to enhance pollen viability [69]. Mo deficiency is reported to have striking effect on pollen formation in maize. Their pollen grains were smaller, free of starch, have much lower invertase activity, and showed very poor germination [22,70].

Generally, Cd strongly inhibits pollen germination and tube growth [9,11,71,72]. However, low concentrations of Cd (10^−12^–10^−10^ M) are reported to have stimulatory effect on growth rate and tube elongation [73], which was attributed to stimulating the enzymatic activities at the low concentrations [9,74]. However, high concentrations of heavy metals inhibit the enzymatic activities and, thus, might constrict the pollen germination. In our study, low concentrations of Cd (0–100 μM) was found to stimulate pollen germination and tube growth, however, the pollen tubes showed a range of strong morphological abnormalities, characterized by irregular or anomalous growth, such as swelling at the tip of the pollen tube and extremely contorted growth. However, stimulation of the germination rate was observed up to 10^−4^ M Cd during the initial (1–2 h) stages of pollen tube growth, which is a relatively high concentration compared to some reports of 10^−10^ M Cd, for *Lilium longiflorum* and *Nicotiana tabacum* pollen grains [73] and ~10^−6^ M Cd for *Plantago depressa* [72]. In the current study, lower concentrations of Cd have greatly affected the plant growth (plant biomasses) and pollen morphology as previously described, while in vitro treatments at higher concentrations up to 100 μM stimulates the pollen germination rate, though it causes some abnormalities for pollen tube, which demonstrates that in the in vitro experiments pollen may be less sensitive to Cd than in vivo. Several studies have correlated the negative effects of the heavy metals on the pollen germination and pollen tube growth to several factors, including enzyme activity decline, preventing DNA replication and protein synthesis in plants which, in turn, affect pollen germination [75]. In *Picea wilsonii*, Cd stress strongly inhibited pollen germination and tube growth by disrupting the endomembrane organelles, inhibiting endo/exocytosis, and forming acidic vacuoles, resulting in swollen tube tips and irregularly broadened tube diameters [13].

Low doses of Se (10 μM) and Mo (1 μM) had a positive effect on the pollen germination and pollen tube growth under Cd stress, which suggest that both Se and Mo can counteract the damage effects of Cd in the in vitro pollen germination. Similar findings have been reported where Se was found to promote the germination of olive pollen in both drought stressed and non-stressed conditions [69]. The authors further checked H_2_O_2_ levels in the pollen under draught stress to conclude that the addition of Se strongly counteracted the ROS accumulation, thus, the amount released from pollen of stressed plants was similar to control plants [69]. Mo has also been described to promote pollen germination at low concentrations in strawberry, but at higher concentrations it was found to decrease it [76]. Similarly, Raohavan and Baruah found that ammonium molybdate stimulated germination and growth of *Areca catechu* pollen even in the absence of boron, which is essential for pollen germination media used in their study [77]. On the other hand, molybdenum was claimed not to have any stimulation effect upon either germination or growth in *Setaria sphacelate* [78]. Thus, the impact of Mo on pollen germination and pollen tube growth might be affected by some other parameters and might strongly depend on the plant species.

Heavy metals uptake and transport in plants are controlled by a group of metal transporters. Manipulation of these transporters activities could confer or weaken the plant tolerance to Cd stress. *IRT1* is responsible for uptake of iron from soil, however it can mediate variety of other heavy metals, including the essential metals such as zinc and manganese as well as toxic metal such as cadmium [79]. In *A. thaliana*, *AtIRT1* has been identified for mediating Cd uptake from soils and could be regulated by overexpression or deletion mutation [80]. In *A. thaliana*, *AtIRT1*-overexpressing plants accumulated more amounts of Cd as compared to wild-type plants [30]. After Cd absorption through the plant roots, it can then be transferred into plant shoots through the xylem, which contribute to most of the Cd accumulation in the plant shoots [81]. In *A. thaliana*, *AtHMA2* and *AtHMA4* pump Cd into the xylem, which is necessary for root-to-shoot Cd translocation [31,32]. Overexpression of *AtHMA4* increased root-to-shoot Cd/Zn-transport and, thus, enhanced the plant metal tolerance [82], whereas disruption of *AtHMA4* function led to hypersensitivity to Cd and Zn in *A. thaliana* [83]. In *Brassica napus*, high expression of *IRT1* in root was responsible for the high Cd uptake of one cultivar (L338), as compared to another one (L351). However, the L351 accumulated more Cd in the plant shoots as compared to L338 cultivar, which was due to the different efficiency of Cd translocation by the xylem. This was further explained as result of the higher expression of *HMA2* and *HMA4*, in L351 as compared to L338 [84].

In our study, Se enhanced the expression of *IRT1* in root tissues leading to enhanced Cd-uptake by the roots, which could explain the high Cd content in Cd + Se treatment in roots as compared to Cd only. The expression of *HMA2* and *HMA4* was significantly reduced under Cd stress, except on applying Se, which up-regulates these genes leading to more root-to-shoot translocation of Cd in a good agreement with the high Cd concentration accumulated in stem under the same treatment. There are several reports in literature indicating that the increased expression level of *HMA2* or *HMA4* induced Cd xylem uploading for translocation to the shoots, leading to higher Cd content in plant shoots [82,85]. In contrast, Mo application down-regulated *IRT1* gene expression resulting in lower Cd uptake by the plant roots. Additionally, it did not enhance genes responsible for Cd-translocation, i.e., *HMA2* and *HMA4*, which interpret the lower accumulation of Cd in plant stems in Cd + Mo treatment.

On the other hand, our results indicate that *HMA3* was induced in the plant tissues under Cd treatment, especially on applying Mo. The overexpression was more pronounced in leaves as compared to roots. Basically, *HMA3* expression level varies from Cd-hyperaccumulators to non-hyperaccumulators. Thus, high expression of this gene is required for Cd hypertolerance in the Cd-hyperaccumulation for sequestering Cd into leaves’ vacuoles, particularly in young leaf cells [86,87]. However, the situation is different in Cd-non-hyperaccumulators, where it displays higher expression levels in the plant root than shoot, resulting in higher Cd concentration in the plant root, and lower root-shoot Cd translocation [87,88]. For instance, in *Oryza sativa*, overexpression of *OsHMA3* reduced the accumulated Cd in grains and enhances the plant tolerance to Cd [89,90]. Therefore, *B. napus*, as Cd-hyperaccumulator, is highly expected to have higher *HMA3* expression in leaf tissues as compared to roots.

Phytochelatins (PCs) are generally known to have a significant role in heavy metal detoxification in plants; either via shuttling PC–Cd complexes into plant cell vacuoles or through the long-distance transport in the root-to-shoot and shoot-to-root directions [35]. PCs are essential for Cd tolerance in plants and all the mutants that are unable to synthesize those peptides are Cd-hypersensitive [91]. Both Se and Mo up-regulate the expression of *PCS1* in the plant tissues, which would enhance the plant tolerance to Cd. This Cd-tolerance enhanced the plant growth even if the Cd content was high, like in the case of Cd + Se treatment, as compared to Cd only. However, applying Se and Mo together down-regulates the expression of *PCS1* which accounts for the plant Cd-sensitivity and the retarded growth. The essential roles of PCs in metal detoxification by plant cells are as known as the Cd-tolerance with the overexpression of PC synthase genes. In *B. napus*, high levels of PCs were detected in the phloem sap when the plant was exposed to Cd stress [35].

In summary, Se and Mo foliar application could alleviate Cd toxicity in *B. napus* and limit its accumulation in the plant seeds which are mainly used as a source of oil. Cd toxicity and the protective role of either Se or Mo could be clearly observed in the pollen grains morphology, fertility, and the in vitro germination rate. The mechanisms by which Se and Mo control Cd reaching the plant seeds is different and it does not depend only on controlling Cd-uptake by the plant root. Metal transporters specially *HMA3* and synthesis of phytochelatins are two key mechanisms the plant utilize to cope with Cd stress.

## 4. Materials and Methods

### 4.1. Soil Properties, Selected Plant Species, and Experimental Setup

#### 4.1.1. Soil Properties

Soil used in this experiment was collected from the test field in Huazhong Agricultural University, Wuhan, China. The soil sample was transferred to the greenhouse of the Micro-element Research Center (30°28′′26′′ N, 114°20′51′′ E, and 30 m above sea level) for grinding and sieving. Soil analyses were performed at the beginning to test different parameters following different methods. Physicochemical characteristics of the soil were as follow: pH 7.12; Organic matter, 13.72 g/kg; available potassium, 193.48 mg/kg; alkaline hydrolysis nitrogen, 52.33 mg/kg; available Se, 0.022 mg/kg; available Mo, 0.068 mg/kg; total Cd, 0.072 mg/kg; and Olsen-P, 16.57 mg/kg.

#### 4.1.2. Selected Plants

The L351 rape genotype of high Cd accumulation ability in shoots and low Cd accumulation in roots was used in this study. In our previous study, 165 different rape lines were selected for pot and hydroponic culture experiments to investigate the Cd concentrations in different parts of rapes under Cd stress to screen high/low Cd accumulating rape lines. Finally, one rape genotype of high Cd accumulation (L351) was used in this experiment [84].

#### 4.1.3. Pot Setup and Monitoring

The polluted soil required for the pot experiment was prepared by mixing the soil with 5 mg·Cd/kg·soil cadmium chloride hemipentahydrate (CdCl_2_ × 2.5 H_2_O, 98%, purity) in aqueous solution. The Cd solution was applied to the soil surface in polyethylene pots (20 cm diameter and 15 cm depth) and thoroughly mixed, then saturated with water, and air dried at room temperature. The wetting-drying mixing process was repeated to ensure soil equilibrium for a two-month period under natural light at a minimum temperature of 10–11 °C, a maximum of 25–30 °C, and a relative humidity of about 30–40%.

The experiment was set up into eight treatments with four replicates, including control, noted as Ck, selenium only (Se), molybdenum only (Mo), both selenium and molybdenum (Se + Mo), Cd only (Cd), Cd with Se (Cd + Se), Cd with Mo (Cd + Mo), and Cd with both Se and Mo (Cd + Se + Mo). In each replicate basal nutrients (N, P, and K) and others microelements were supplemented with a given and measured quantity. During fertilizers application, the soil in the pots was covered with polythene bags to avoid the entrance of either fertilizer to the soil.

The seeds were sown in each pot and the seedlings were thinned to two per pot. The seedlings were allowed to grow for 30 days in the pots for acclimatization before foliar spray of fertilizers to be applied. The foliar application of Se and Mo was carried out using 1.0 mg Se L^−1^ (as sodium selenite (NaSeO_3_) and 0.3 mg L^−1^ Mo (as ammonium molybdate [(NH_4_)_6_Mo_7_O_24_·4H_2_O]) solutions. The spraying was performed in the morning on a dry and sunny day and was repeated two times a week for ten weeks. The same amount of distilled water was used for control experiment. At the beginning stage of flowering, the florets (1–3 cm) were sampled out from both the control and treatments groups. At the same stage, three-plants of every treatment were harvested, roots and leaves were separated, washed, and immersed in liquid nitrogen, and then stored at −80 °C for total RNA extraction. At the end of the plant growth period, the roots of all the plants were separated from their shoot and rinsed with tap water to remove adhering soils before being washed with deionized water. All roots were soaked into 20 mmol/L EDTA-Na_2_ solution for 10 min to remove metal ions from root surfaces. Plant samples were then dried at 65 °C for one week until they reached a constant weight. Then the dried plant materials were weighed and ground.

### 4.2. Pollen Grain Studies

#### 4.2.1. Development

For the characterization of heavy metal effects on pollen developmental stages, anthers, flowers, and young buds were removed from the plants under different treatments. The specimens were fixed in FAA (formaldehyde, glacial acetic acid, and 70% ethanol, 5:5:90), stored in 70% ethanol, embedded in paraffin, and then sectioned at 8 μm with a Leitz 1512 microtome. Staining was carried out with Mayer’s hematoxylin-eosin method [92], and at least 20 flowers were studied under a light microscope (BX51; Olympus, Tokyo, Japan), and differences between pollen grains collected from polluted and non-polluted samples were analyzed.

#### 4.2.2. Morphology by SEM

For thorough studies of pollen grains morphology, samples were collected from mature flowers and studied using scanning electron microscope (SEM). Tissues were critical-point dried with liquid CO_2_, set on aluminum stubs with two-sided tape, coated with gold (Edwards S150B Sputter Coater, BOC Edwards, UK), and observed with a JEOL JSM-6390/LV SEM (JEOL, Japan) at 20 kV.

#### 4.2.3. Fertility Using I_2_/KI Solution

Flowers from polluted and unpolluted samples were collected, anthers were dissected out and prefixed in 70% ethanol, then the flowers were placed on a glass slide and the sliced anthers were opened with forceps until they released pollen grains. Then the pollen was stained with 1.0% I_2_/KI solution (1–2 drops on each slide), incubated for 5 min, then observed and photographed. Well-stained (black) and perfect pollen grains were considered as fertile, while the immature pollen grains appeared orange to red [93].

#### 4.2.4. In Vitro Germination

To study the protective role of Se and Mo on the in vitro pollen germination rate and pollen tube growth, similar treatments to those in the in vivo experiment were planned. Thus, pollen grains, after hydration, were sown in an aqueous culture medium containing 5% sucrose and 20% PEG 4000 [94]. To investigate the metal influence on germination rate, the medium also contained 0 (control) or 10–500 μM cadmium, applied as chloride salt (CdCl_2_ × 2.5 H_2_O), without/with added Se (1, 10, and 20 μM), Mo (1, 5 and 10 μM), or both at the same time. Pollen grains were collected from freshly dehisced anthers on a dry slide and exposed to vapor-phase pre-hydration for 20–30 min in Petri plates lined with moist filter paper. Hydrated pollen grains were cultured in sitting drops (20 μL) of germination media, then the slides were incubated at room temperature for 90 min. Germination rate (%) was calculated based on protuberance of the pollen tube by observing 100 randomly selected pollen grains in each replicate. A pollen grain was considered germinated if the pollen tube length was more than twice the pollen grain diameter [95].

### 4.3. Analytical Tests

To determine total metal content in plant tissues, aqua regia method was used [96]. Hence, approximately 0.5 g of ground plant sample was digested using 10 mL of a HNO_3_/HClO_4_ (9:1) mixture at a constant temperature of 200 °C. The sample was heated to the point of white smoke from perchloric acid. The digested solution was cooled and diluted to 25 mL using deionized water. The diluted solution was filtered and Cd concentrations were measured using atomic absorption spectroscopy (Z-2000, HITACHI, Japan). A standard material of plant GBW10015 (GSB-6) was used for the quality assurance and quality control of Cd analytical procedure with recovery rate of 90 ± 10%.

For Se concentration measurement, 0.5 g of a plant sample was digested following the same method described above. However, after heating to the point of white smoke from perchloric acid, 10 mL of HCl and water (1:1, *v*/*v*) were added, and then heating continue until white smoke appeared again. The sample was then diluted to 25 mL using deionized water and filtrated. The obtained solution was used for Se determination by dual-hydride generation atomic fluorescence spectrometry (AFS-9700, Haiguang, China) following the hydride generation-atomic fluorescence method (GB/T21729. 2008) with the same standard material used for Cd.

Mo determination was carried out according to the procedure described by Wan et al. [97]. Thus, 0.3 g of the plant sample was dry-ashed at 550 °C for 8 h. Mo was then measured by the polarographic catalytic wave analysis method using a JP-4000 oscilloscope polarograph in a reaction solution (2 mL 2.5 mol/L sulfuric acid, 1 mL 0.5 mol/L benzo hydroxy acetic acid, and 5 mL saturated sodium chlorate solution).

### 4.4. Total RNA Extraction and Quantitative RT-PCR

Frozen plant tissues were utilized for the total RNA extraction using TRIzol reagent (Invitrogen, Carlsbad, CA, USA). The quality and the concentration of the extracted RNA were assessed using 1.0% agarose gel and NanoDrop 2000 spectrophotometer (Thermo Scientific, Waltham, MA, USA), respectively, thereafter, it was stored at −70 °C until further use. Quantitative real-time PCR was carried out using an IQ5 Multicolor RT-PCR Detection System (Bio-Rad, Hercules, CA, USA) with SYBR Green Master (ROX) (Newbio Industry, China) according to the protocol provided by the manufacturer at a total reaction volume of 20 μL [98]. The thermal cycling conditions were; 95 °C for 3 min, 40 cycles of amplification (95 °C for 10 s, 60 or 58 °C for 30 s, and 72 °C for 20 s), and a final extension at 65 °C for 1 min. Gene-specific primers for BnActin (as a reference gene) and for the other investigated genes (*IRT1*, *HMA2*, *HMA3*, *HMA4*, and *PCS1*) were designed using the primer designing tools of IDTdna (http://www.idtdna.com, access on 19 April 2018), as listed in Table S1. The gene expression was normalized using *BnActin* as a reference gene. The relative expression levels were calculated by comparing the cycle thresholds (CTs) of the target genes with that of the reference gene *BnActin* using the 2^−ΔΔ*C*t^ method [99,100]. The quantified data were analyzed using the Bio-Rad IQ 5 Multicolor Real-Time Manager software. Finally, the relative expression levels of different genes were detected. RT-PCR experiments were performed on three biological replicates, with three technical replicates for each sample.

### 4.5. Statistical Analysis

All the data were subjected to the two-way analysis of variance (ANOVA) using SPSS 22 software (SPSS inc., Chicago, IL, USA). Mean values of each treatment were compared using the Duncan test at the *p* < 0.05 level. Figures display the mean and standard error of the data. Graphs were plotted using SigmaPlot 12.0 Software.

## 5. Conclusions

This study has identified foliar application of Mo and Se to affect their mutual absorption and accumulation in different plant tissues with a pronounced negative impact on *B. napus* growth in case of their combined application at the used dosages. Moreover, Mo and Se application could control Cd uptake, and its further accumulation in different parts, specially seeds, in the oilseed rape, with a minimum seed content of Cd in Cd + Mo treatment. Foliar spraying of Se or Mo fertilizers seems to provide a protection role on pollen grains in *B. napus* when growing in Cd polluted soil. Furthermore, our results explain how Se and Mo could control Cd-content in seeds. While Se enhances the Cd-uptake in *B. napus*, it limits its accumulation in seeds via manipulating the expression level of *PCS1* gene, which is responsible for phytochelatin production that drives Cd sequestration into plant cell vacuoles instead of having them interfere with cellular processes. On the other hand, Mo originally down-regulates the expression of *IRT1* responsible for metal-uptake, and it further up-regulates *HMA3*, mediating Cd efflux into leaves’ vacuoles to finally control the accumulated Cd in seeds. Our study does not recommend the combined application of Se and Mo as foliar fertilizers for *B. napus* due to their negative effect on plant growth, even in non-polluted soil. However, further studies are still required to investigate the other possible techniques, such as seed soaking or seed additives, or the use of different doses of these fertilizers when applied as foliar spray.

## Supplementary Materials

Supplementary materials can be found at http://www.mdpi.com/1422-0067/19/8/2163/s1.
