# Supplementary material for: Can Selenium and Molybdenum Restrain Cadmium Toxicity to Pollen Grains in Brassica napus?
Source: Int J Mol Sci. 2018 Jul 24;19(8):2163. doi: 10.3390/ijms19082163 (PMC6121452; doi:10.3390/ijms19082163)
Supplement: Supplementary file 1 [file ijms-19-02163-s001.pdf]

Article

Can Selenium and Molybdenum Restrain Cadmium Toxicity to Pollen Grains in *Brassica napus*?

Marwa A. Ismael [1,2,3], Ali Mohamed Elyamine [1,2], Yuan Yuan Zhao [1,2], Mohamed G. Moussa [1,2,4], Muhammad Shoaib Rana [1,2], Javaria Afzal [1,2], Muhammad Imran [1,2], Xiao Hu Zhao [1,2] and Cheng Xiao Hu [1,2,*]

[1] Key Laboratory of Arable Land Conservation (Middle and Lower Reaches of Yangtze River), Ministry of Agriculture, Huazhong Agricultural University, Wuhan 430070, China; maf02@fayoum.edu.eg (M.A.I.); elyoh@hotmail.fr (A.M.E.); yuanyuanzhao666@gmail.com (Y.Y.Z.); MohamedGomaa_Ali@agr.asu.edu.eg (M.G.M.); muhammadshoaib@webmail.hzau.edu.cn (M.S.R.); juvaria_afzal@outlook.com (J.A.); imrangorayauaf@yahoo.com (M.I.); xhzhaowhu@163.com (X.H.Z.); hucx@mail.hzau.edu.cn (C.X.H.)

[2] Hubei Provincial Engineering Laboratory for New-Type Fertilizers, Huazhong Agricultural University, Wuhan 430070, China

[3] Botany Department, Faculty of Science, Fayoum University, Fayoum 63514, Egypt

[4] Soil and Water Research Department, Nuclear Research Center, Egyptian Atomic Energy Authority, 13759, Abou Zaabl, Egypt.

* Correspondence: hucx@mail.hzau.edu.cn

Table S1. Sequences of primers used for RT-PCR

Target	Primer sequences (5`–3`)	Annealing Temperature	Gene IDs
BnActin2.1	F-CTCTTTCACACGCCATCCTCC<br>R-GATTCCAGCAGCTTCCATTCC	57.5 C	
IRT1	F- GCCTGATTCTTTCGAGATGT<br>R- TGCATGCTTGCTAGTGTATAG	58 °C	BnaA03g44160D
HMA2	F- CTTTGGCTACGGCTGATATT<br>R- CATTCTCCACCACTTTCCTT	58 °C	BnaC07g50890D
HMA3	F-GGCTGGAGTTTACCCTATTC<br>R-CCAATCTGCAACCGAGAA	58 °C	BnaA01g06890D
HMA4	F-GCAGTCACTGATGAGCTTAG<br>R-TTTCCATCCACCACAATACC	58 °C	BnaC03g48480D
PCS1	F-GACCACCATTGACGACTTT<br>R-GCCATATCTCTCTCAGCATTAT	58 °C	BnaA06g36340D

Note: ‘F’ was defined as forward primer, and ‘R’ as reverse primer.
